# Novel greener approached synthesis of polyacrylic nanoparticles for therapy and care of gestational diabetes

**DOI:** 10.1080/10717544.2020.1809555

**Published:** 2020-09-03

**Authors:** Xianghong Cheng, Yahui Xu, Qian Jia, Ning Guo, Zhenzhen Wang, Yu Wang

**Affiliations:** Department of Obstetrics, School of Clinical Medicine, Henan Provincial People’s Hospital, People's Hospital of Zhengzhou University, Henan University, Zhengzhou, China

**Keywords:** *Ramulus mori* extract, polyacrylic, Au nanoparticles, gestational diabetes mellitus, drug delivery, nursing care

## Abstract

In the present medical diagnostic method for the therapeutic of gestational diabetes mellitus (GDM), it is problematic and difficult to release successful and secure release of drugs to the exact site. Hence, many researchers have been carried out to bring antidiabetic using modern method to release of drugs for their production. This research work focusses on to provide an assemblage to the recent growth in the field of *Ramulus mori* extract (RME) loaded on polyacrylic gold nanoparticle for antidiabetics with special highlighting on nursing of GDM. Keynote of gold nanoparticle: diabetes mellitus, nursing, insulin, antidiabetic, drugs, and new system for drug delivery. Rat is used to test the drug delivery system. *In vivo* examination was not prepared seldom including in this research paper. This research investigation could be a new avenue for the development of drug delivery system of GDM.

## Graphical Abstract

**Figure:**
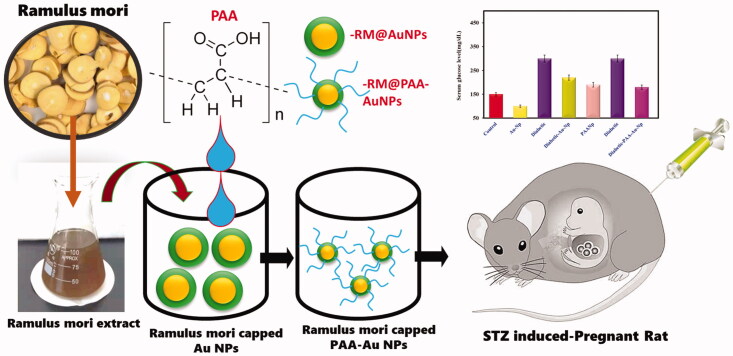

## 1. Introduction

Diabetes mellitus (DM) disorder is the metabolic change in the blood glucose (hyperglycemia). Presently, more than 415 million people suffer from diabetes in the world, and the number will be increased to 642 million people in 2040. The main medical symptoms of DM are reported such as high emaciation, fatigue, body weakness, fat looking and particularly polydipsia problems. In many cases of severe DM, it leads to the unadorned kidney failure, cardiac failures, vision problems, and amputation (Anushka & Mahomooda, 2014). The three types of main diabetes are type 1, type 2, and gestational diabetes. For the first type, it was well known as insulin-reliant or juvenile diabetes, type 1 diabetes, also known as insulin-reliant or juvenile diabetes, consequences to produce deficiency of insulin due to the T-cell mediated deficiency of insulin due to the T-cell-mediated damage of its secretion base, were the type 2 diabetes involves the lack of ability of cells in the liver, muscles, and adipose tissue to react to the usual functions of insulin. Patients in type 1 are characterized by hyperglycemia and hyperinsulinemia, usually lacking insulin conflict. Though patients with type 2 diabetes are characterized by insulin conflict, which revenue their reaction to insulin blunted, accordingly affecting hyperglycemia. Gestational diabetes occurs for the period of pregnancy due to insufficient insulin response, which is usually gone after the baby birth (Ben Haroush et al., 2004; Metzger et al., 2007).

The previous reports elaborated that primary main factors of gestational diabetes are an insufficient insulin secretion during pregnancy periods. The causes of gestational diabetes mellitus (GDM) have been demonstrated by three different regions: (1) dysfunction of autoimmune cells, weakened insulin secretion level after infiltrate genetic abnormalities and mainly cell dysfunction is significantly connected with enduring insulin opposition. Insulin secretion conflicts and impairments have been rised through the above three factors, as well as significant changes in the secretion mechanisms of hormones and cortical from human placenta lactogen. In addition, hormones of progesterone and estrogen also provide noteworthy disturbance of body glucose and insulin stability (Gilmartin et al., 2008). Though, the clear evidence of molecular mechanisms and genetic models on pancreatic cells that connected to insulin secretion level in the production of GDM is still not reported. The occurrence of GDM is predictable to be around 15% worldwide. This is estimated to increase due to gradually increasing body weight of women as well as fatness in their reproductive age. The nursing supervision of GDM is required in terms of way of life modification (exercise, diet, and nutrition) and improved medication. Adherence is vital to avoid maternal and neonatal perinatal mortality (Mensah et al., 2019). The systemic analysis of retroactive cohort system was used to examine pregnancy women by two step method of Coustan’s criteria (C&C). Generally, pregnancy periods of all women are 28 weeks except women have pre-pregnancy DM. Many logistic deteriorations were applied to improve therapeutic potential in GDM therapy associated with numerous risk factors. These risk factors of GDM well-defined by C&C method is pre-pregnancy body mass index reported as more than 24.2 kg/m^2^ (Hung et al., 2018). The connotation between interpregnancy interval and gestational diabetes was studied by pregnant mothers using conditional logistic worsening, which has been elaborated that pregnancies greatly matching to the same mother and attuned risk factors that changes in mother diagonally pregnancies. In previous reports, a comparative study was directed between GDM mothers with adjusted odd ratio of 1.1 (95% CI) for time of 24–59 months and 1.51 (95% CI) for time of 110 months or more months, which has been compared with 18–23 months of IPI. Those results demonstrated that petite IPI (about 6 months) upsurges the risk factors of gestational diabetes and propose that detected investigative results in previous researches might be varied between corresponding mothers (Gebremedhin et al., 2019). Mulberry leaves (*Ramulus mori*) contain huge amount of oxyresveratrols. Traditional medicine has been used in China, due to their antidiabetic nature. In this study, we observed that RME exerted an inhibitor effect on the lipopolysaccharide induced production of the pro inflammatory cytokine interleukin-6 in the macrophage cells. Moreover, RME inhibited lipopolysaccharide production by blocking the leukotriene B4 receptor. It depends on the NADPH oxidase 1 and NOX1 reactive oxygen species case capade leading to antidiabetic activity (Park et al., 2016). *Ramulus mori* contains a variety of chemical constituents, which are mainly flavonoids, alkaloids, triterpenoids, polysaccharides, coumarins, etc. (Yang et al., 2014; Dai et al., 2015; Sánchez-Salcedo et al., 2015). In this study, *Ramulus mori* was extracted, and its antidiabetic effect was investigated. the bioavailability of metformin hydrochloride (MH) was developed by oral route. The lactic acidosis is a severe metabolic difficulty that is arised due to the significant accretion of MH during therapy of DM. Forty spans of cholesterol were taken for the MH encapsulated noisome preparation through the method of reverse phase evaporation technique. The MH-encapsulated noisome material was reported to efficiently provide sustained and controlled release of drug molecules, predominantly with positive charged noisome. The bioactivity and biocompatibility of the prepared MH-loaded noisome were examined through study of glucose serum values and metformin value presented in animal models. The reported results exhibited that MH encapsulated noisome has extensive activity and extended releasing properties with hypoglycemic efficiency (Hasan et al., 2013). Nonetheless, therapeutic potential for GDM treatment has some difficulties and troubles for sustained delivery on specific targeted site treatments. Therefore, development of sustained and effective targeted delivery system to deliver anti-diabetics is required for the commercialization. This investigation report was designed to develop innovative delivery system by nanoformulations for delivery of anti-diabetic drug molecules with special accent on vesicular and miscellaneous system (Rai et al., 2016).

In the recent years, nanotechnology grown tremendous development in every field. It has the capacity to work at the atomic, molecular, supramolecular level (on the scale of 1–100 nm). For the study of new material creation, structure and fundamental properties with the functions of resulting their small structure were studied (Roco et al., 2000). Research and robotic technology are expected to make vital development in the mainstream of biomedical applications, including the area of genetic engineering to produce novel drug discovery in the treatment of disease like diabetes, cancer. There is some restriction using traditionally available drug release system. Data regarding particularly some specific target, altered effects and diminished potency due to the drug metabolism in the body, cytotoxicity of assured anticarcinogenic of pharmacological agents were revealed less. Biocompatible nanoparticle with their specific physical, chemical, and biological properties can defeat these boundaries and serve as effective drug delivery systems. This modified drug delivery system has significant advantage over traditionally available drug release system (Subramani et al., 2012). The increasing use of nanoparticle in consumable product and biomedical application leads to improved increase in human contact to engineered nanomaterial (Linkov et al., 2008; Fadeel et al., 2010; Oberdorster, 2010; Teli et al., 2010). This study reveals gestational diabetes management using chitosan encapsulation with bioactive compounds (resveratrol). This one is a substantial way to raise the stability and effectiveness of GDM substances. Synthesis of resveratrol–zinc oxide complex is encapsulated with chitosan (CS–ZnO–RS). This could be used to deliver the resveratrol with minimized side effect and bioavailability. The synthesized complex was confirmed by various techniques such as TEM, SEM, and AFM. The average size of the particle was 38 nm. The drug releasing profile showed that 95% of RS is released from CS–ZnO–RS complex within 24 h. *In vitro* studies confirmed that, highest inhibition of α-glucosidase (77.32%) and α-amylase 78.4% was observed at 500 μg/mL. It showed significant decrease in the blood glucose levels in GDM induced rats and maintained the lipid content toward the normal rats (Du et al., 2020). In the biomedical research introducing nano metal oxide like gold nano rods, iron oxide, TiO_2_, silver, and platinum have been used as carrier in drug delivery system (Frank et al., 2012; Li et al., 2013).

## 2. Materials and methods

### 2.1. Materials

Polyacrylic acid, methanol, gold nano particle (Au), FBS, and other biological mediums were obtained from Sigma Ltd. (Shanghai, China). Double distilled water was used through out all the experiment and solvent utilization.

### 2.2. Methanol extraction of *Ramulus mori* leaf

**Figure 1.**
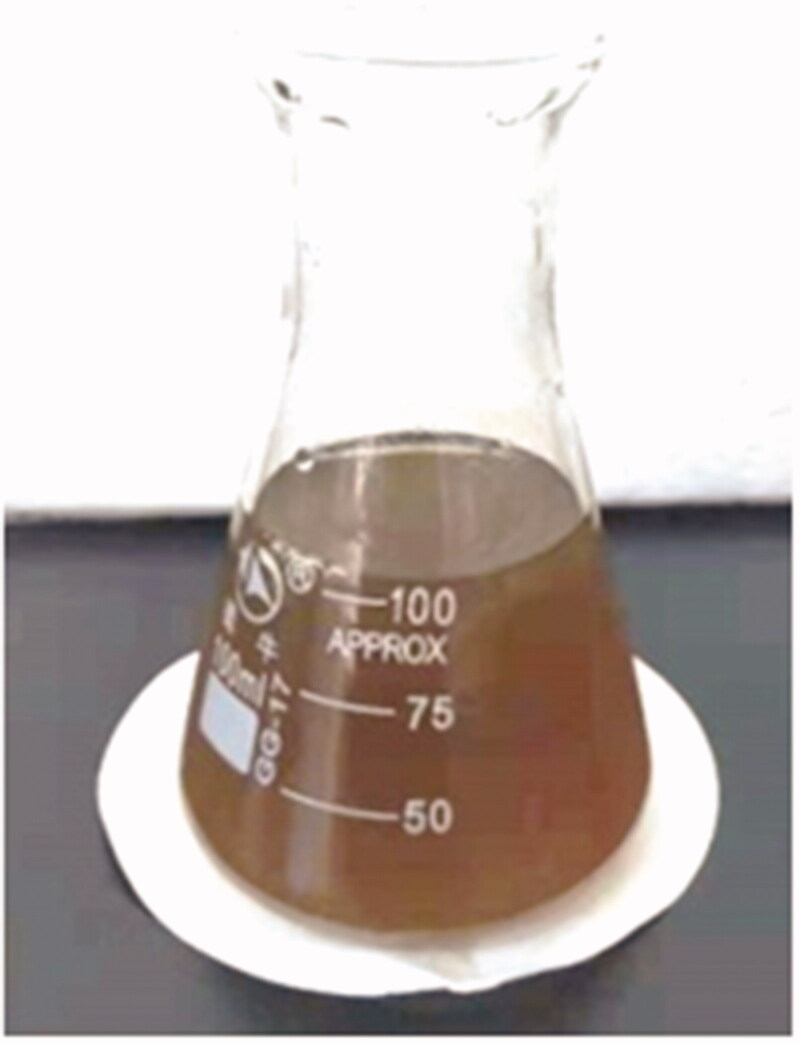
Photo images of prepared *Ramulus mori* methanolic extract.

The leaves of *Ramulus mori* were collected from Henan (Zhengzhou, China). It was washed through water and dried with hot air oven about 1–2 hours at 30 °C. Two hundred milliliters of methanol was taken in a beaker. The leaves were added into the solution stirring carried out at 25 °C for four hours. The extract becomes greenish yellow in color. It was filtered and used for future experiments for gold nanoparticle synthesis (Figure 1).

### 2.3. Synthesis of polyacrylic gold nano particles (Au–PAA)

Six milliliters of AuCl_3_ were mixed with 20 ml of leaf extract in a conical flask. Constant stirring was carried out for four hours at 20 °C. In this stage, polyacrylic acid was introduced to the above solution, stirring continuously about two hours with 40 °C. The color changed green to yellowish green. It indicates the gold nanoparticle was incorporated in the polyacrylic matrix. It was confirmed in the XRD analysis.

### 2.4. Animal preparation method

All animal experiments have been performed under laws and regulation guides of National Institutes of health and Guide for the Care and Use of Laboratory Animals. The use of animals has been approved by the Institution's Animal Care and Use Committee (IACUC) of Henan University, China. Male and female rats were obtained from yellow river breeding laboratories, Zhengzhou, to utilize as GDM representation as reported earlier (Kaufmann et al., 1981; Zou et al., 2018). For the investigational intend, female rats were arbitrarily separated into three testing groups such as, control, Au-Np, and PAA-Np group. The animals were acclimated for three weeks before matting. Individual animal was exclusively recognized by ear punch enclose card. The specific temperature is maintained at 20 °C ± 4 °C with 20–40% moisture. The animals were maintained with three stages: 12 h photoperiod, 12 h of light, and then 12 h of darkness. The rats were fed with chow die containing 28% protein, 46% carbohydrate, and 16% fat (Envigo’s Teklad, ‎Indianapolis, IN‎). Matting was predicted with the examination of copulation plug, which showed gestation (GD) 0. Female is arbitrarily assigned to treatment groups instantly after matting and independently housed in polycarbonate cage like square box (29 cm × 19 cm × 13 cm) with hardwood bedding. The rats were given with Teklad LM-485 rodent diet (Harlan Teklad, Madison, WI) and tap water ad libitum was carried out in the whole study. All the protocol performed on the animals were repeated and agreed by the University of Alabama’s Institution Animal Care and Use Committee (IACUC) (Cima, 2013).

### 2.5. Induction of diabetes

Experimentally, rats were induced to diabetes with single intraperitoneal injection of 70 mg/kg streptozotocin (STZ) of 0.07 mol/L citrate buffer (pH 4.5) on the 6th day after gestation for constantly two days and injected with in five minutes after dissolution (Povoski et al., 1993). Physiological saline was treated to control group rats as vehicle. For the analysis blood glucose level, maternal hyperglycemia was determined. Lower than 200 mg/dL of blood glucose level, animals were preferred for this study.

### 2.6. Dosage of nanoparticle NPs

Mated female rats were investigated randomly to the following analysis: (1) control group, six dose distilled water (*n* = 12), (2) one dose (10 mg NPs/kg body mass) Au NPs (*n* = 14) on the rat, (3) one dose (10 mg Nps/kg body mass) PAA-Np (*n* = 18) on the rat, and (4) six dose (10 mg NPs/kg body mass) Au–PAA-Np (*n* = 18) on the rats. All the doses of nanoparticle were 10 mg/kg body mass which equates to 2.3 mg Au/kg body mass. Ferumoxtran-10 is an intravenous iron oxide Np used in MRI in many clinical studies (Harisinghani et al., 2007; Weissleder et al., 2010). Similarly, the dose of 10 mg Np/kg body mass (3.4 mg Au/kg body mass) was chosen and it corresponds that approximate gold oxide NPs would receive due to undergoing MRI. For the completion of mating period, the male rats were euthanized. For the clinical study, they were monitored daily. When the female was weighted on GD 0, as well as before each dosing, treatments were delivered by insertional injections during gestation. The grouping of animals was (2) and (3) which were maintained a single dose of test material on GD 9, when compared to group (4) which was dosage level of 0.02 mL/g body weight. The control group received an equivalent volume of vehicle (H_2_O) (Table 1).

**Table 1.** Treatment group count of rats in each group (*n*).

	Treatments	*n*
Control	6 dose of distilled H_2_O given on GD 9	12
Au-Np loaded group	1 dose of (10 mg Np/kg body mass) Au-Np given for GD 9	14
PAA-Np loaded group	1 dose (10 mg Np/kg body mass) Au-Np given for GD 9	18
Au–PAA-Np loaded group	6 dose of (10 mg Np/kg body mass) Au–PAA-Np given for GD 9	18

### 2.7. Study on body weight, serum glucose, and insulin

**Figure 2.**
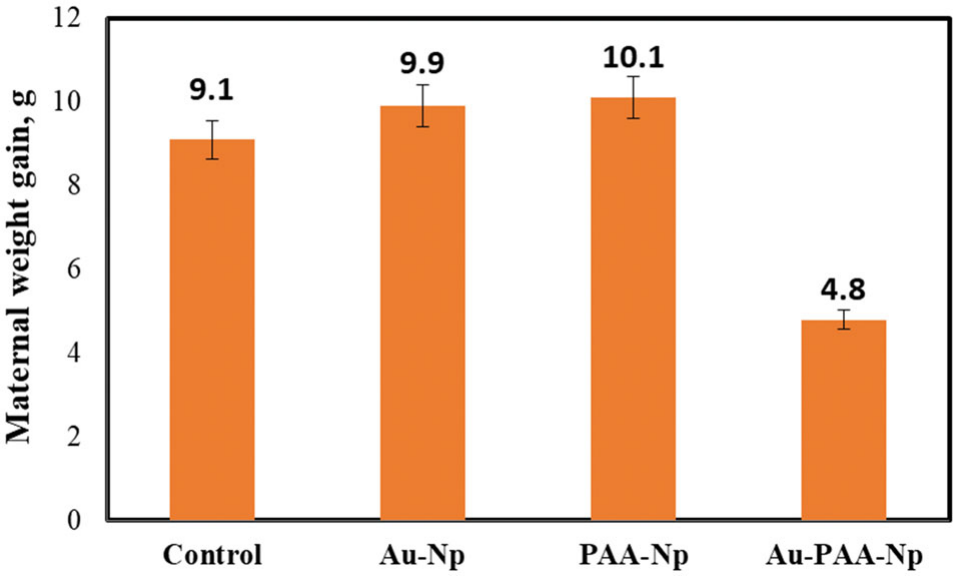
Maternal weight gain analysis by subtracting the female body mass measured on GD 0 from the final body mass minus gravid uteri on GD 16, *n* = 12–18, moderate differences compared to all other groups (*p* < 0.03).

In the period of pregnancy, maternal body weight gain is the sign of gestation. It can have prolonged effects on the developing fetus (Han et al., 2019). Low single dose Np was given to GD9 treatments on groups 1 and 2. It had no reaction on maternal weight gain. About 20% (*p*≤.03), maternal weight gain significantly decreased during gestation, when the animals received the group 3 Au–PAA-Np dosage for nine consecutive days. The comparison to the control group indicates an apparent treatment effect in Figure 2.

### 2.8. Fetal analysis of pregnant rats

**Figure 3.**
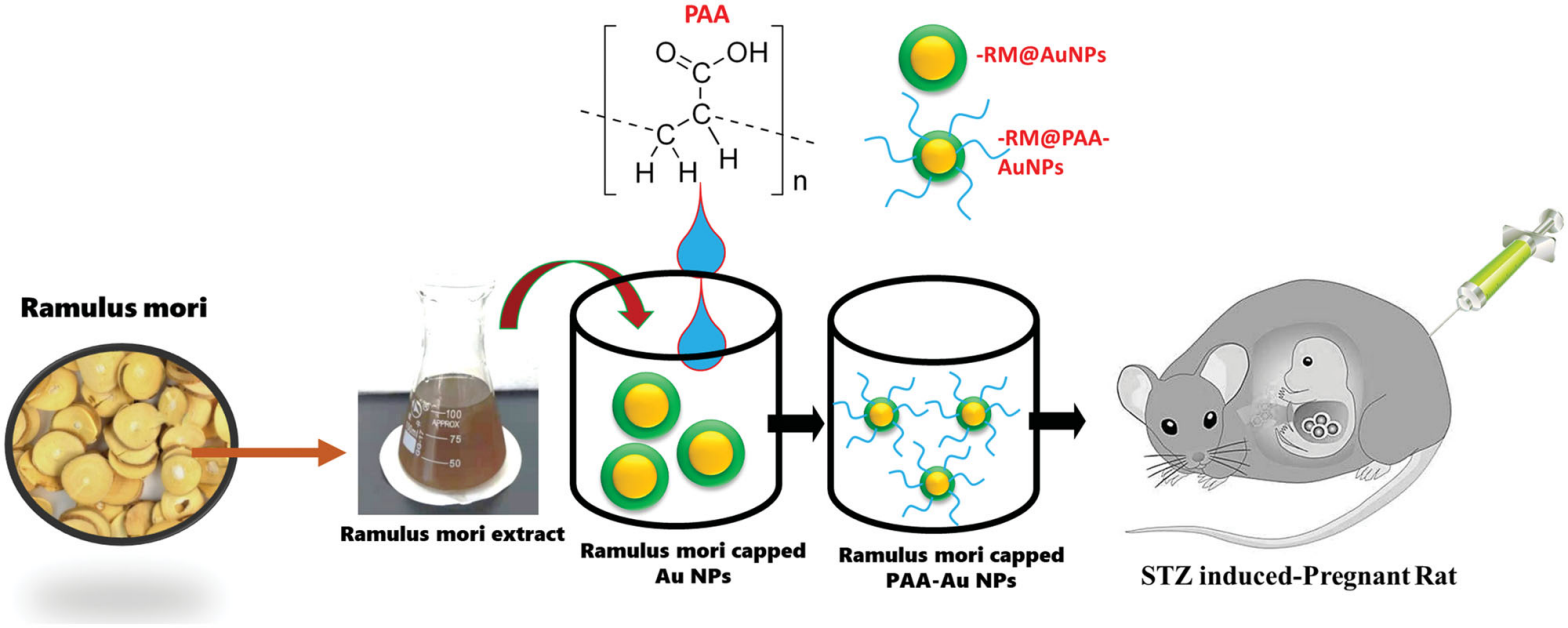
Schematic representation of the present investigation.

The pregnant rats were given to the ketamine–xylazine for anesthetic. After that, it was evaluated on GD18 by cardiac puncture with liver cut. After the cesarean section, the size of the litter was counted in grouping. Viable fetuses were identified by virtue of their insulin level and weight. Serum glucose was analyzed for further study (Figure 3).

### 2.9. Statistical analysis

The statistical analysis was done with multiple replicates. Pregnant female rats were used for the experimental unit of this analysis. The data for each replicate was intended separately, tested for the homogeneity of variance. Further mean of level statistics was analyzed using SPSS (SPSS Inc., Chicago, IL) and then pooled and analyzed to give the reports that are shown in Table 2. Data were analyzed by one-way analysis of variance (ANOVA).

**Table 2.** Maternal weight gain (g ± SEM) for the analyzed groups.

Maternal weight gain, g	Control	Au-Np	PAA-Np	Au–PAA-Np
	9.1 ± 0.7	9.9 ± 0.9	10.1 ± 0.3	4.8 ± 0.6

## 3. Results and discussion

Optical observation was characterized with Shimadzu 2400 UV-visible spectrophotometer (Kyoto, Japan) operated at a resolution of 1 nm. An Advance diffractometer (BrukerD8, Billerica, MA) was used to determine the X-ray diffraction (XRD) pattern of synthesized Au–PAA composite. The composite was diluted with suitable solvent and placed a drop on the surface of the copper grid for the HR-TEM investigation. The functional group was studied using Jasco-FT-IR spectroscopy (Oklahoma City, OK).

**Figure 4.**
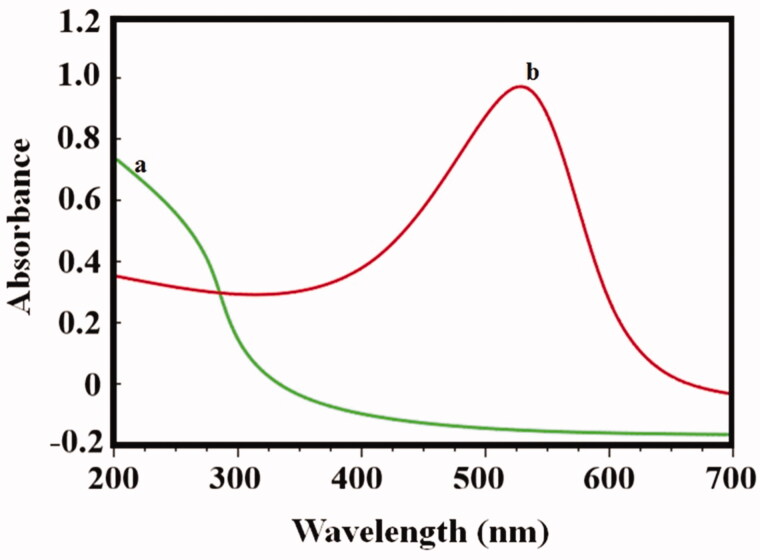
UV spectra of (a) pure Au and (b) plant extracted PAA composite.

**Figure 5.**
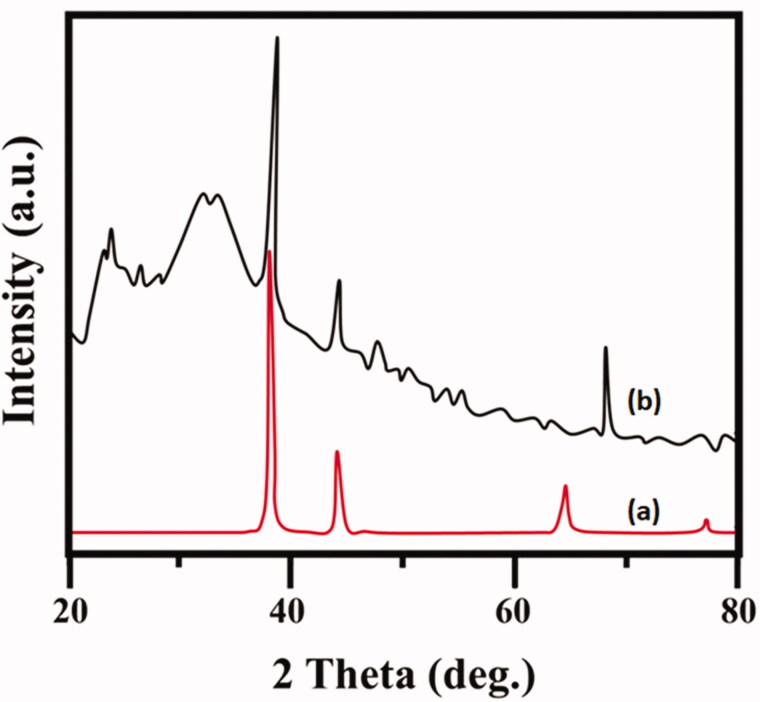
XRD diffraction of plant extracted Au–PAA NPs composite.

**Figure 6.**
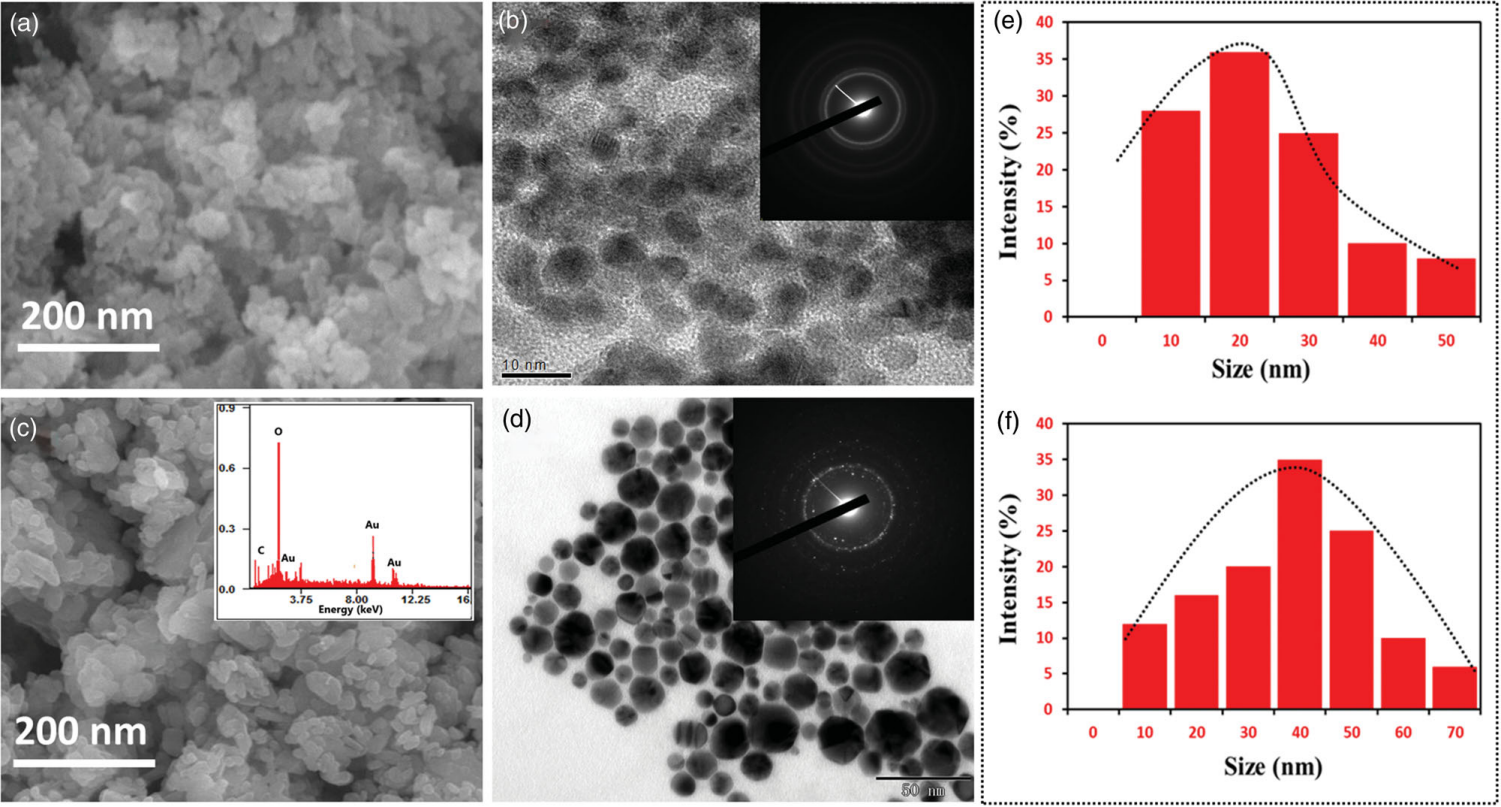
FE-SEM and HR-TEM images of synthesized (a, b) Au-NPs (c, d) Au–PAA-NPs in the plant extract; DLS size analysis of synthesized Au NPs under extracts.

The absorption of Au–PAA synthesis by using *Ramulus mori* extract (RME) is shown. UV spectrum (Figure 4(a)) indicated that *Ramulus* extract contains little protein from the small shoulder near 272 nm. The formation of Au–PAA is completed in two hours, which is identified by the color change accordingly. The spectrum of Au–PAA absorption showed larger band around 545 nm (4b). This is interrelated to the cluster–polymer interactions. This intense absorption band is due to the surface coating of nano gold particles, which arise due to polyacrylic acid on the surface electrons of Au (Yajie et al., 2018). The XRD in Figure 5 shows that gold particle is present in four identical peaks at 2*θ* = 36.0, 43.1, 63.4, and 76.3. These peaks corresponded to standard Bragg reflections plane of (111), (200), (220), and (311) of face center cubic (fcc) lattice (Sneha et al., 2014). It indicates fcc structure of gold confirmed in Au–PAA composite. Figure 6 shows the FE-SEM and HRTEM images of synthesized Au NPs and Au–PAA with RME. The image showed the formation of gold particle in PAA on the matrix. The shape of the PAA surface with Au is shown spherical in shape and with the particle size 22 nm, which has been confirmed by DLS analysis as exhibited (Figure 6(f)). The formation of nano composite with gold nanoparticles is observed in HR-TEM images.

**Figure 7.**
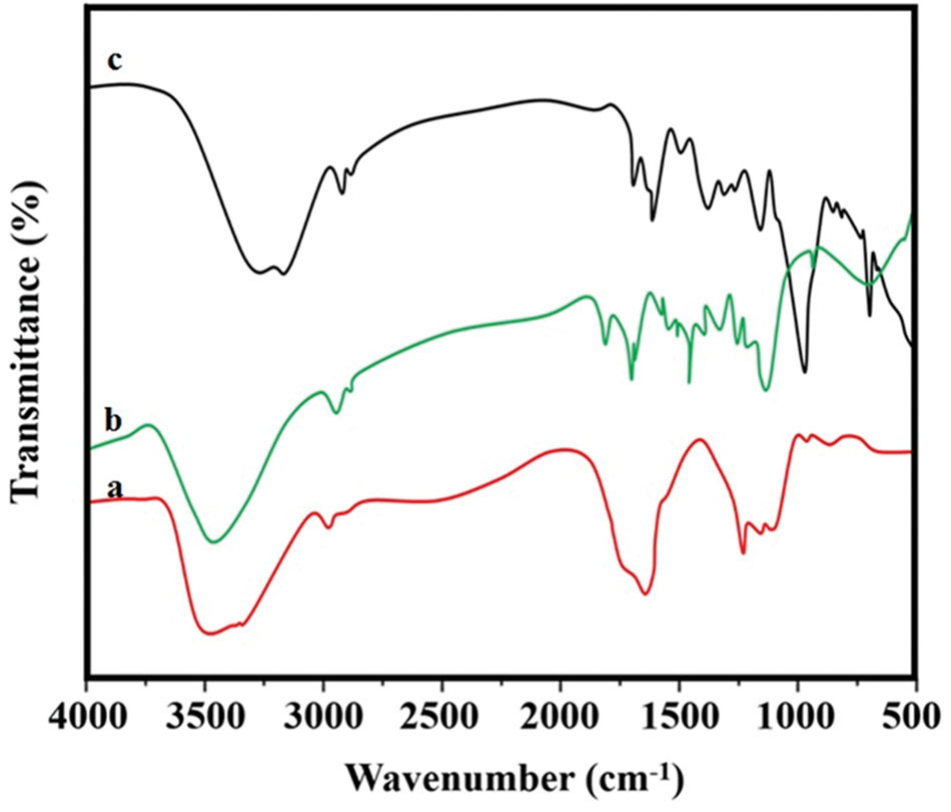
FT-IR spectra of (a) plant extract *Ramulus mori*, (b) pure PAA, and (c) PAA-Au composite.

Figure 7 shows the FT-IR images of functional group identification of the RME with Au–PAA component involving in the capping and reduction of Au nanoparticles. Peaks at 2950 cm^−1^, 1734 cm^−1^, and 1452 cm^−1^ indicate the presence of carbonyl group present in the PAA spectra. The peak showed the transmittance at 1641 cm^−1^, 2085 cm^−1^, 3452 cm^−1^, and 638 cm^−1^ (Figure 7(a)). The stitching vibration bands show plant extract at 3448 cm^−1^, 2919 cm^−1^, 1729 cm^−1^, and 1630 cm^−1^ which conformed the composite formation (Figure 7(b)). It reveals the C–C carbonyl bond, O–H stretch, and C–H bonds of methanol solution leaf extract. The reduction of AuCl_3_ into Au nanoparticle was confirmed by the vibration spectra found in Figure 7(b). When Au–PAA-Np showed the clear shift in the spectrum of transmittance, this is due to the interaction of nanoparticle with the plant extract biomolecular that acts as stabilizing agent (Lishuai et al., 2019).

**Figure 8.**
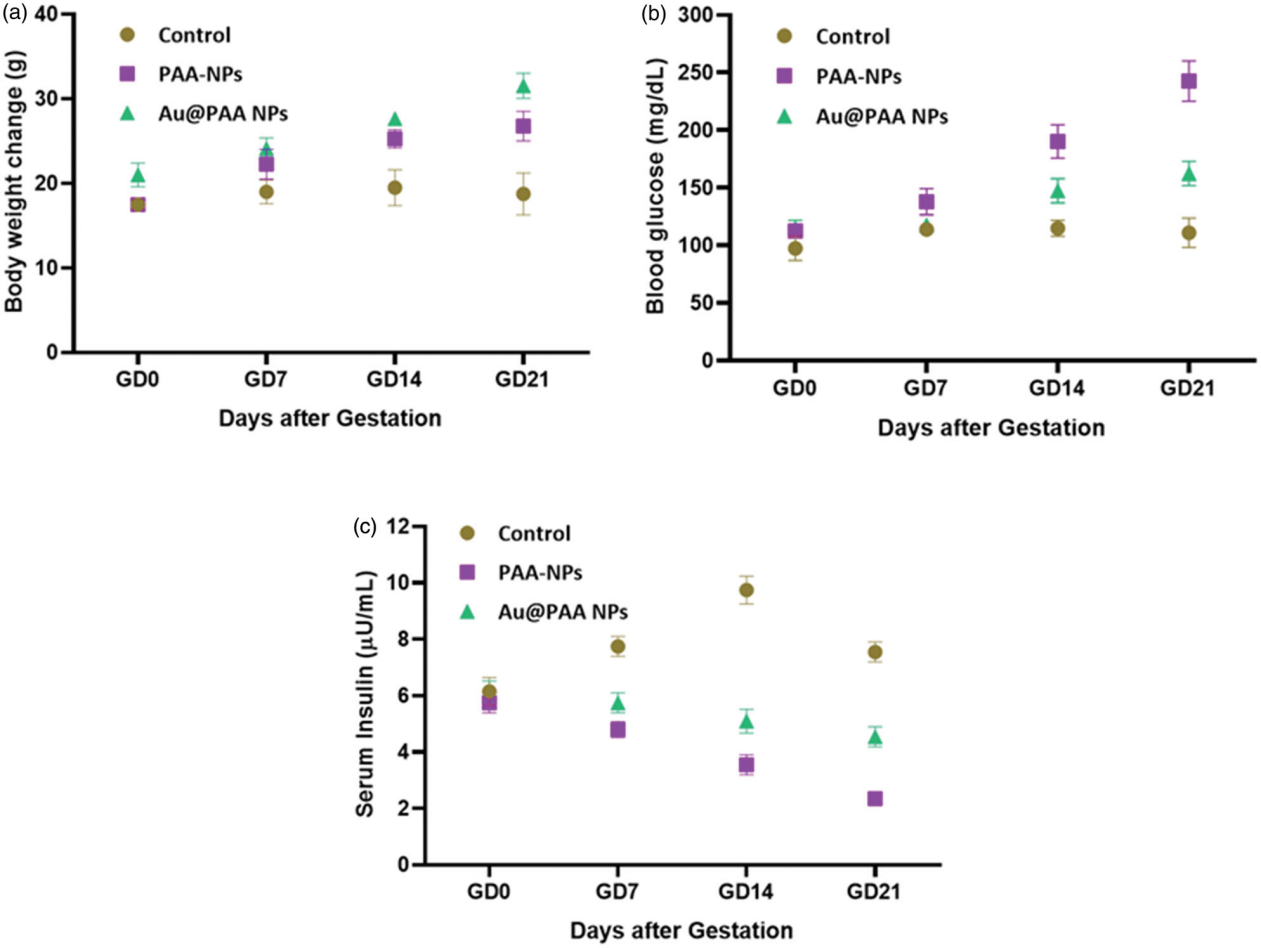
Biological and chemical evaluation of levels of serum glucose in female rats in the experimental and control groups.

Many risk challenges are associated with the development of GDM which includes superior maternal age, maternal obesity, high parity, previous delivery of macrocosmic baby, family record of type 2 DM, stature of short maternal, polycystic ovary disease. It leads to high levels of saturated fat in diet. In prior GDM, neonatal death, prior cesarean delivery, some of the other risk factors were previous birth or congenital malformations and high blood pressure during pregnancy, several pregnancies (Ashwal & Hod, 2015). The levels of glucose in rats were evaluated. It showed the glucose level (mg/dL) reached at highest point. When it is compared with diabetes alone or with Au-Np and PAA-Np, by the executive with the leaves of AuNPs, it decreased the glucose levels, while it was greater than the normal glucose level either in diabetes alone or were combined with PAA. The graphical value showed the significant decrease glucose level in Au–PAA Np dosed rats (Figure 8).

**Figure 9.**
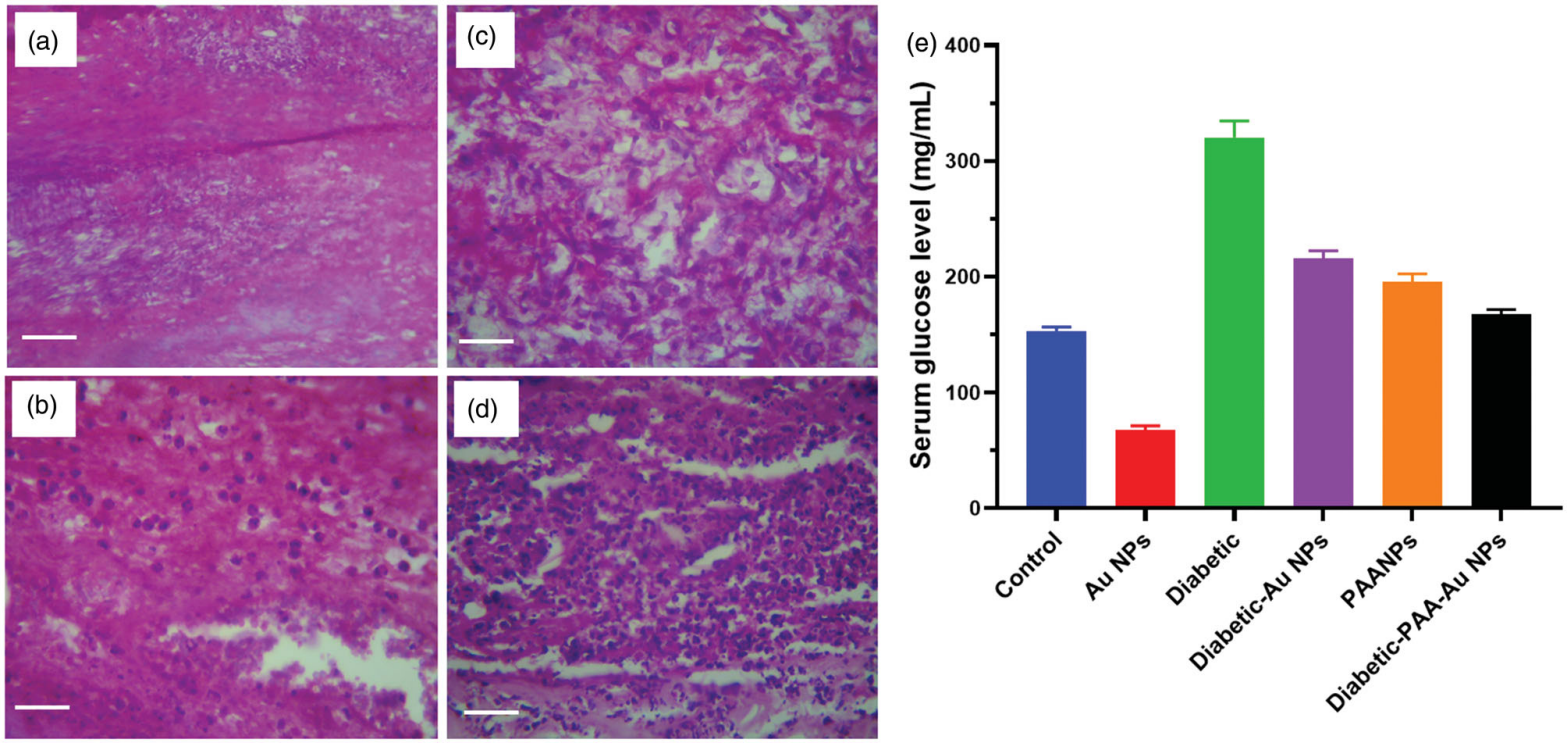
The stained fetal liver of gold content using emerald blue (red indicates the presence of Au) in the grouping (a) control H_2_O treated, (b) 1 dose of Au–PAA-NPs, and (c) 8 dose of Au–PAA NPs.

Gold concentration was considered in the sample taken from the mother fetus to establish if nanoparticle was capable to cross the placenta into the fetus. There is not much difference occurred in gold concentration in the liver of fetuses from dams dosed Au–PAA-Np colloid on GD9 through GD16 compared to the untreated controls. In addition, rats received only one dose of nanoparticle with colloid. There is no gold observation in the fetal liver samples. There is significant increase in gold nano particle which were experiential in fetal liver and placenta in the animal treated with *Ramulus mori* with Au–PAA for nine consecutive doses. It is not done with other treatment group (Figure 9). In this figure, red color particle showed the presence of gold nanoparticle. When the observation of increasing the dosage of gold concentration with the leaf extract in rats, a sharp increase is shown in Figure 9. When they are treated with multiple doses of Au–PAA-NPs, they were used contrast agents of MRI due to their aptitude to deposit in organs such as kidney, spleen, and lymph nodes. The study is like biodistribution which were reported earlier (Khurana et al., 2012).

## 4. Conclusions

This study finds greener synthesis of gold nanoparticle (Au-Np) using *Ramulus mori* leaf extract using methanol in an ecofriendly approach. PAA–Au was synthesized using institute chemical polymerization in the presence of leaf extract. XRD pattern results confirmed the presence of well dispersed Au nanoparticles in the matrix of the polymer. UV–vis analyses of leaf extract were compared with PAA Np. It confirmed the extract reduced the gold iron. From the FT-IR, it reveals the formation of extract Au–PAA-Np. The carboxylic groups of acrylic acid were found to be conscientious of gold ions mobility in the polymeric matrix. TEM images conformed the particle size of gold nanoparticle (nm). Microscopic observation on diabetic mother rats showed typical changes in liver cell layers. But the rat’s liver received Au NPs in the maternal level, which exhibited notable amelioration. In addition to biochemical assessment exposing that the application of Au–PAA-NPs affects the amelioration of the changes in Au serum glucose concentration in maternal part. This study reported that AuNPs were active against diabetic. This study proposed a novel method for the treatment of GDM.
